# Self-reported consumption frequency of meat and fish products among young adults in Kazakhstan

**DOI:** 10.1177/02601060221114230

**Published:** 2022-07-13

**Authors:** Venera Akhmetova, Yuriy Balji, Yelena Kandalina, Ainara Iskineyeva, Akmaral Mukhamejanova, Akmaral Baspakova, Yassin Uzakov, Kuralay Issayeva, Galia Zamaratskaia

**Affiliations:** 1 Department of Food Technology and Processing Products, S. Seifullin Kazakh Agro Technical University, Nur-Sultan, Kazakhstan; 2 Department of Veterinary Sanitation, 374659 S. Seifullin Kazakh Agro Technical University, Nur-Sultan, Kazakhstan; 3 Department of Foreign Philology, 326804 A.Baitursynov Kostanay Regional University, Kostanay, Kazakhstan; 4 Department of Biotechnology, 186034 Toraighyrov University, Pavlodar, Kazakhstan; 5 Department for Scientific Work, 186040 West Kazakhstan Marat Ospanov Medical University, Aktobe, Kazakhstan; 6 Department of Food Technology, 373812 Almaty Technological University, Almaty, Kazakhstan; 7 Department of Molecular Sciences, 8095 Swedish University of Agricultural Sciences, Uppsala, Sweden

**Keywords:** Meat consumption, fish consumption, university students, young adults, food

## Abstract

**Background:** Meat and dairy products are important ingredients in Kazakhstan, although there are indications that high consumption of red and processed meat is associated with a risk of several non-communicable diseases and has an adverse impact on the environment. **Aim:** The aim of this study was to investigate the dietary habits of young adults in Kazakhstan, particularly meat and fish consumption frequency among university students in five regions of Kazakhstan. **Methods:** The assessment of meat and fish consumption was based on the food frequency questionnaire. Region of residence, age, sex, weight, height and parental education were also self-reported. **Results:** Meat consumption among the participants was lower than recommended consumption of 1500 g per week in Kazakhstan but almost two-fold higher than the World Cancer Research Fund recommendations of 500 g per week. Approximately 24% of the participants reported to consume meat every day. Only 8.6% of the participants reported fish consumption in line with the recommendation of approximately 270 g per week in Kazakhstan. Meat and fish consumption was fairly homogeneous across regions and sex. **Conclusion:** The results from this study contribute to the relatively limited information on meat and fish consumption in Kazakhstan. Further knowledge on dietary habits and probably improved nutrition recommendations on meat consumption in Kazakhstan are needed to protect public health and the environment.

## Introduction

Diet plays an important role in our health and welfare, and interest in healthy eating is growing. In Kazakhstan, meat and dairy products are important ingredients in traditional cuisine. During 1992–2000, the most consumed animal-derived foods were milk (75%), followed by meat (20%) and eggs (below 5%) (Liang et al., 2020).

Nowadays, the role of meat and meat products in human diet is extensively discussed. Over the last decade, it was repeatedly suggested that high consumption of red and processed meat is associated with an increased risk of several non-communicable diseases including type 2 diabetes mellitus (DM), cardiovascular disease and some forms of cancer (Wolk, 2017). Moreover, increasing global meat production leads to an adverse impact on the environment. Thus, there is a general trend to reduce meat consumption, especially red meat, and substitute it at least partly with chicken meat and plant-based analogues (Banovic and Sveinsdóttir, 2021; Collier et al., 2021).

Whereas the total fish consumption in the world has increased over the past 50 years, some countries of the former Soviet Union tended to consume less fish (Supartini et al., 2018). Fish consumption is associated with healthy eating because fish is the main dietary source of the n-3 long-chain polyunsaturated fatty acids (n-3 PUFA) and also contain vitamin D, a number of minerals and high-quality protein (Chen et al., 2022). In contrast to some other Asian countries, inhabitants in Kazakhstan consume more meat than fish (York and Gossard, 2004). For example, fish consumption in children aged 9-10 years constituted 2% of total protein intake, whereas meat – 43% (Sharmanov et al., 2018).

Unhealthy eating including overconsumption of fast food, sugar and salty snacks, is common among adolescents and young adults (De Vet et al., 2015). This might lead to an increased risk of the development of chronic conditions. Investing in adolescent and young adult healthy eating can reduce the risks of development of non-communicable diseases in the future. Consumption of animal-based products among young people in grassland countries or regions is however not well studied.

The aim of this study was to investigate the dietary habits of young adults in Kazakhstan. Specifically, we focused on the frequency of meat and fish consumption among university students in different regions of Kazakhstan.

## Materials and methods

### Consumption frequency and socio-demographic factors

The assessment of meat and fish consumption was based on the food frequency questionnaire (in Russian). Following food items related to the consumption of fresh red meat, processed meat, chicken, and fish were included: fresh meat intake included beef, pork, horsemeat, lamb, goat, and poultry, boiled or fried; processed meat items included sausages and salami; fish and seafood consumption included salmon types, carps, and perch types, as well as molluscs and crustaceans. Answer “other types” were also included. For each food item, the participants were asked to select the following frequency: 1) every day; 2) 4–6 times per week; 3) 2-3 times per week; 4) ones per week; 5) 2-3 times per month; 6) ones per month; 7) less than ones per month; 8) never. Then, the answers were categorized into three categories: i) 4–7 times per week; ii) 1–3 times per week; iii) less than once per week. Consumption quantity per week was estimated as (reported times per week * 150) as each serving of unprocessed meat and fish in Kazakhstan is 150 g. Alcohol intake and smoking habits were not considered in this study. A questionnaire was pretested by senior researchers from the Central and North regions before using it in the survey.

**Figure 1.**
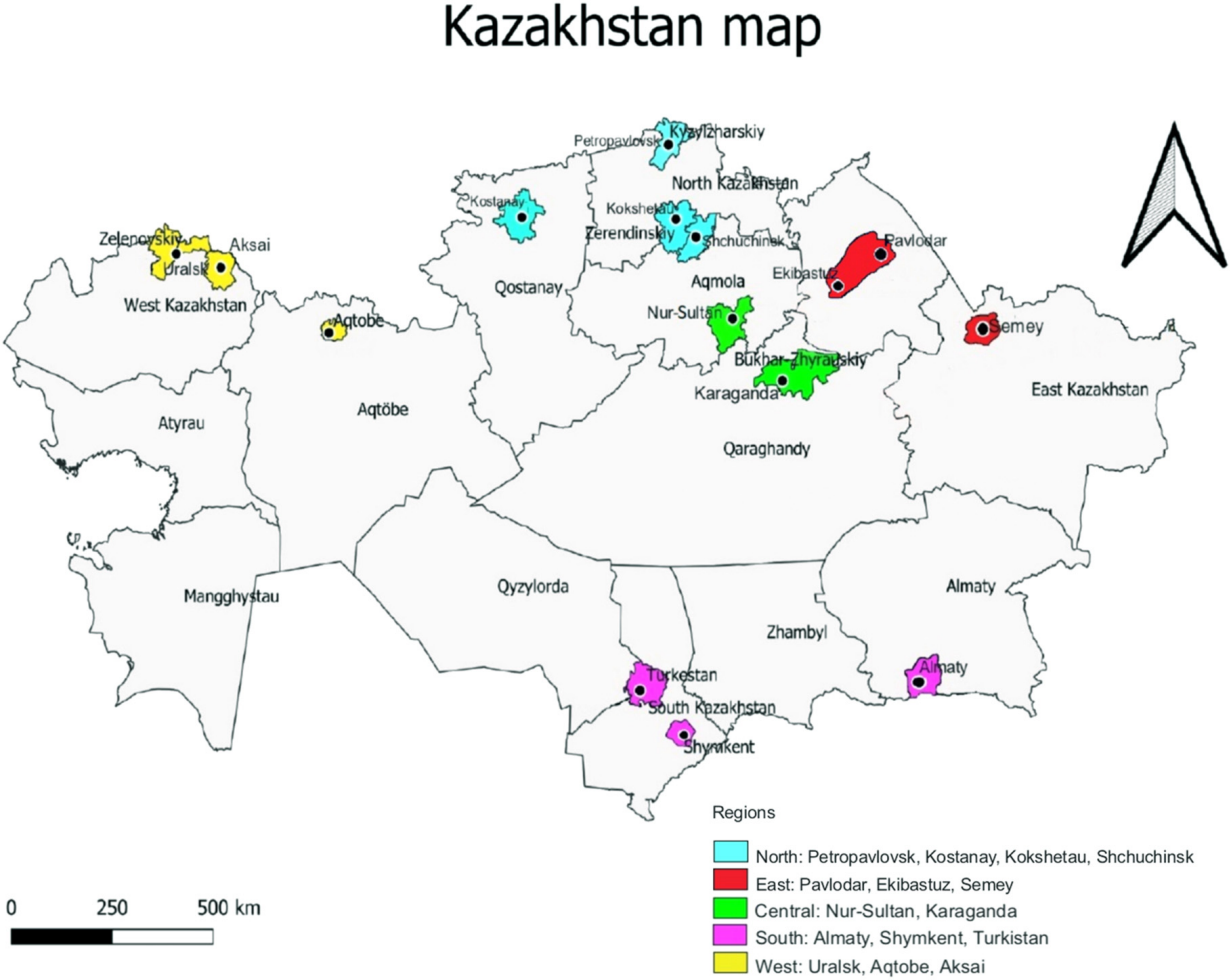
Map of Kazakhstan with the regions where meat and fish consumptions were assessed.

Region of residence, age, gender, height, weight, and parental education were also self-reported. The following regions were included: North (cities Kostanay, Petropavlovsk, Kokshetau, Shchuchinsk); West (Aksai, Uralsk, Aktobe), Central (Nur-Sultan, Karaganda), South (Almaty, Turkistan, Shymkent), and East (Pavlodar, Semey, Ekibastuz) (Figure 1). Body mass index (BMI) was calculated from self-reported weight and height, and weight status was categorized as i) under-weight (<18.5 kg/m2), ii) normal weight (18.5–24.9 kg/m2), iii) overweight (25.0–29.9 kg/m2) and iv) obese (≥30 kg/m2).

### Participants and informed consent

A cross-sectional study was conducted over the largest universities in five regions of Kazakhstan (Figure 1). The study protocol was approved by the local commission on bioethics of the West Kazakhstan Medical University named after Marat Ospanov, meeting number 4, protocol 4, assigned number 16/1 (8 April 2021). Requests to complete the online food frequency consumption questionnaires were sent out to the students of by e-mail and via WhatsApp groups. The participants were informed about the purpose and duration of the study, data protection, and data retention. The present study included questions about food consumption and did not include the processing of sensitive personal data. Participation in the study was voluntary and anonymous. No responses to the questionnaires used in this study include information that can be traced to, or used to identify any individual. Data were collected in May 2021.

A total of 1069 young adults participated in the study. Of these, 83 participants (7.6%) failed to fill in all the data required. The answers from 986 participants at the age of 16 to 30 years were included in the study.

### Statistical analysis

Data were analysed with SAS Version 9.4 (SAS Institute Inc., Cary, NC, USA). Descriptive statistics were used to determine absolute and relative frequencies of categorical variables. Bivariate associations of the self-reported fish and meat frequency consumption (percentages) with the region, sex, fathers’ and mothers’ education were estimated using a series of chi-square tests. Then, differences in calculated consumption quantity per week were evaluated using the mixed model with the fixed effect of regions, sex, fathers’ and mothers’ education. Weight category and age were not included in the final model due to lack of significance and lack of any changes in the model outcomes. Association between BMI, and meat and fish consumption frequencies was estimated using the mixed model with the fixed effect of regions, sex, fathers’ and mothers’ education, and meat type and fish consumption frequencies. The level of statistical significance was set at p < 0.05.

## Results

### Characteristics of the study population and frequency of meat and fish consumption

The main socio-demographic characteristics of the participants are presented in Table 1. The highest number of answers were collected from the Central region of Kazakhstan (39%). Among the participants, there were more women (76%). Further, 17% of participants were categorized as underweight and 12% were overweight or obese.

**Table 1.** Demographic characteristics of the study sample (n = 986).

		Number	Percentage
Age, years	20 or below	512	52
	Above 20	474	48
Sex	Male	234	24
	Female	572	76
Region	East	127	13
	North	113	12
	Center	388	39
	South	172	17
	West	184	19
Weight status	Underweight	164	17
	Normal weight	696	71
	Overweight	102	10
	Obesity	18	2
Mother's education	Primary school	12	1
	College or trade school	528	54
	University degree	446	45
Father's education	Primary school	25	2
	College or trade school	599	61
	University degree	362	37

**Figure 2.**
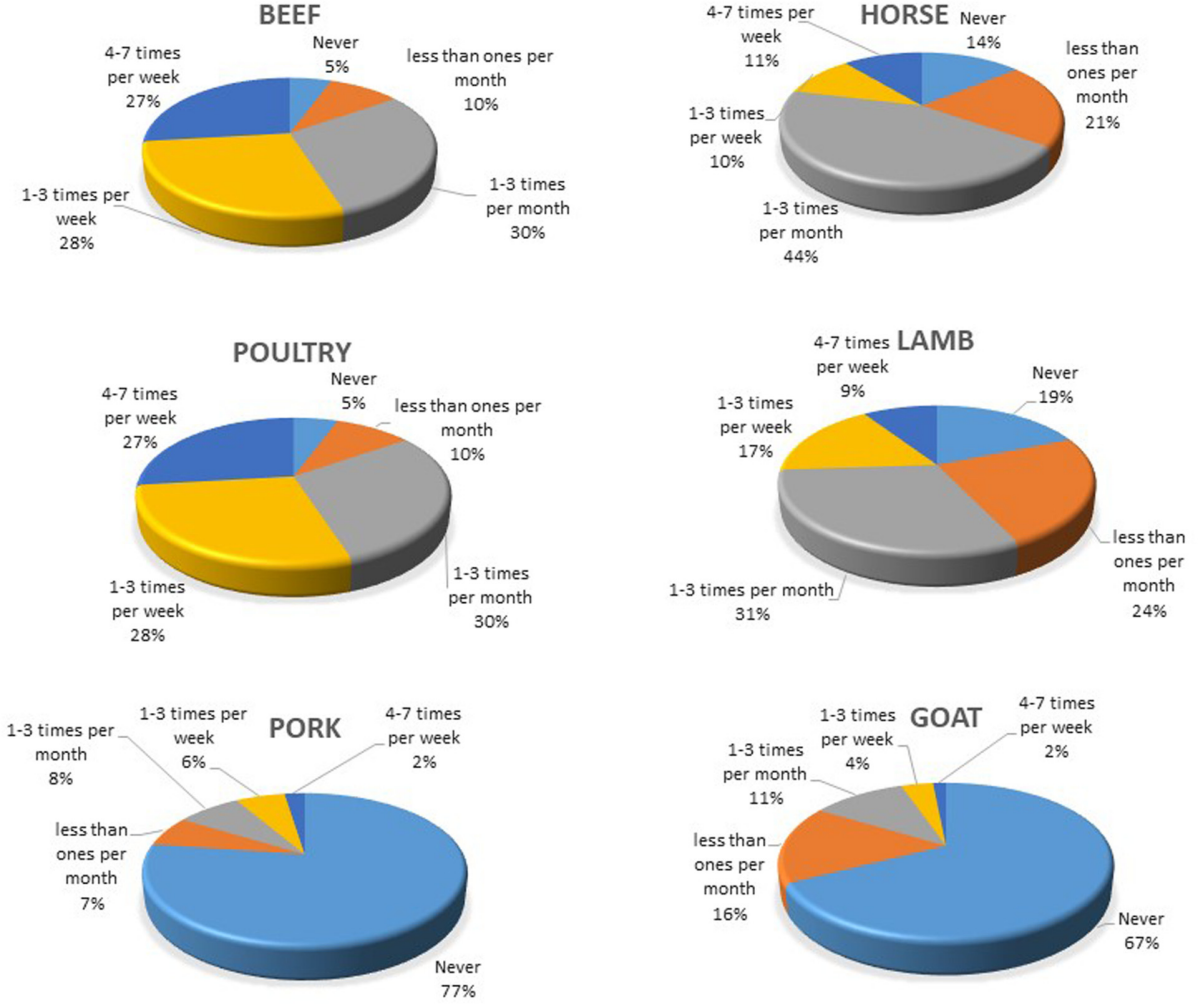
Self-reported consumption frequency of beef, poultry, pork, lamb, horse and goat meat among young adults in five regions of Kazakhstan (n=986).

**Figure 3.**
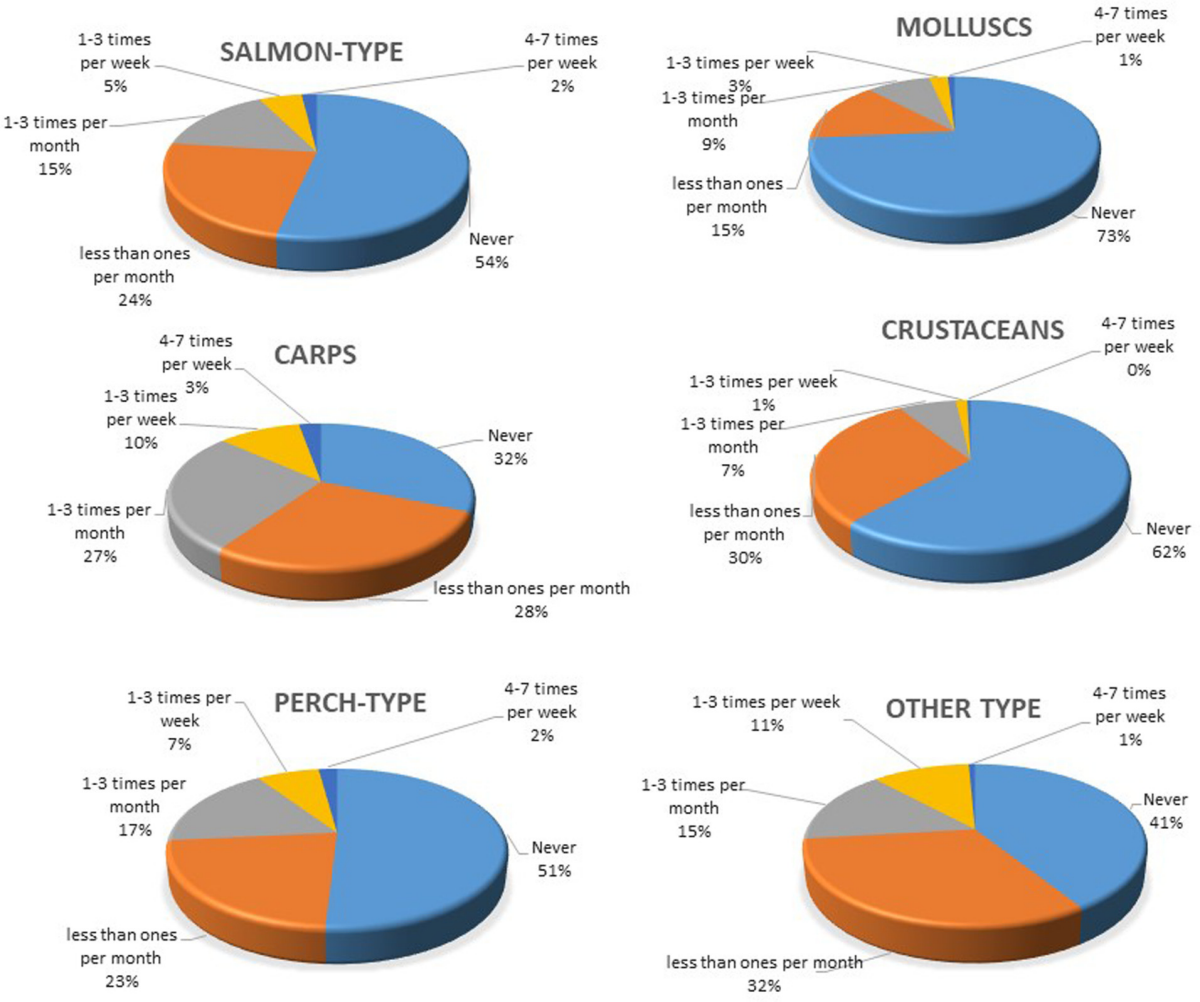
Self-reported consumption frequency of salmon, perch, carp, molluscs, crustaceans and other types among young adults in five regions of Kazakhstan (n=986).

Among 986 participants, six (0.6%) never eat meat, and 24% of the participants eat meat every day. Reported frequencies of consumption of different meat and fish type are presented in Figures 2 and 3, respectively. The most commonly consumed meat-types were beef and poultry, followed by horse and lamb (Figure 2). Pork and goat meats were less consumed. On average, the participants consumed beef 2.4 times per week, poultry – 1.6 times per week, horse and lamb – ones per week, pork – 0.3 times per week, and goat – 0.2 times per week. It appears that 77% and 67% of these surveyed never ate pork and goat, respectively. Boiled meat was consumed slightly more often, 1.8 times per week, than fried meat, 1.5 times per week (data not shown).

Generally, fish consumption frequency was lower compared to meat. Among the participants, 88 (9%) never eat fish and seafood, and 77% of the participants eat fish and seafood more seldom than once per month. Only 8.6% of the participants in our study reported fish consumption frequency of 2-3 times per week. The frequency of carp consumption, 0.5 times per week, was greater compared to the consumption of other types of fish (Figure 3).

### Effect of socio-demographic characteristics on meat and fish consumption frequency

Meat and fish consumption frequencies per sex, parental education, and region are presented in Table 2. According to the chi-test, beef consumption frequency did not differ between the regions (p = 0.085) and sex (p = 0.186) and was not associated with either mother's (p = 0.177) or father's (p = 0.438) education (Table 2). Poultry consumption was highest in the East and Central Kazakhstan (p = 0.038). No sex-associated differences were observed in poultry consumption frequency (p = 0.927). Mother's education was not related to poultry consumption frequency (p = 0.985), although poultry consumption frequency was lowest among participants with fathers who have a university degree (p = 0.038). Horse consumption frequency tended to be higher in the East and Central Kazakhstan (p = 0.053). Male participants were less likely to consume horse meat more than 4–7 times per week compared to female participants (p = 0.007). Neither mother's (p = 0.232) nor father's (p = 0.774) educations were associated with horse consumption frequency. Lamb consumption frequency did not differ between the regions (p = 0.118) and was not associated with mother's (p = 0.325) and father's (p = 0.073) education. Female participants were more likely to consume lamb 4–7 times per week compared to male participants (p = 0.009). Goat consumption frequency was lowest in the North Kazakhstan (p = 0.022). No sex-associated differences were observed in goat consumption frequency (p = 0.944). Mother's education was not related to goat consumption frequency (p = 0.136), although the frequency was lowest among participants with fathers with a primary school education (p = 0.037). Pork consumption frequency was highest in the North Kazakhstan (p = 0.005). Pork consumption did not differ between sexes (p = 0.282), and was not associated with mother's education (p = 0.564). Pork consumption frequency was higher among participants with fathers with a primary school education (p = 0.007).

**Table 2.** Bivariate associations of the self-reported frequency consumption of fish and meat with demographic factors.

Variable		Sex		Mothers' education			Fathers' education			Regions				
		Male	Female	Primary school school	College or trade	University degree	Primary school	College or trade	University degree	East	North	Center	South	West
	Consumption*													
Beef, %	High	23	28	25	26	28	20	25	30	28	23	29	30	22
	Moderate	32	27	0	28	29	24	28	28	24	27	32	22	29
	Low	45	45	75	46	43	56	46	42	47	50	39	48	49
	p-value	0.186		0.177			0.438			0.085				
Poultry, %	High	21	21	17	21	21	24	18	26	14	14	24	18	26
	Moderate	11	12	8	12	11	4	12	10	17	10	10	12	11
	Low	69	68	75	67	68	72	70	64	69	76	66	70	63
	p-value	0.927		0.985			0.038			0.038				
Horse, %	High	6	13	17	11	11	16	11	11	7	11	11	9	16
	Moderate	13	9	8	8	13	16	10	11	11	7	13	7	9
	Low	81	78	75	81	76	68	79	78	82	82	76	84	75
	p-value	0.007		0.232			0.774			0.053				
Lamb, %	High	6	10	17	11	7	16	9	10	7	7	11	10	8
	Moderate	23	16	17	17	18	28	19	14	24	13	20	12	16
	Low	72	74	66	72	75	56	72	76	69	80	69	78	76
	p-value	0.009		0.325			0.073			0.118				
Goat, %	High	2	2	8	2	1	8	1	2	1	0	2	2	2
	Moderate	5	4	8	5	3	4	5	3	10	2	5	2	4
	Low	93	94	84	93	96	88	94	95	89	98	93	96	94
	p-value	0.944		0.136			0.037			0.022				
Pork, %	High	3	2	8	3	2	12	3	1	1	7	2	2	1
	Moderate	8	5	0	6	6	0	6	7	5	12	5	5	7
	Low	89	93	92	91	92	88	91	92	94	81	93	93	92
	p-value	0.282		0.564			0.007			0.005				
Fish, %	High	3	5	8	14	5	12	4	5	6	2	6	3	4
	Moderate	20	17	0	19	16	0	16	21	19	19	17	17	19
	Low	77	78	92	76	78	88	80	73	75	79	77	80	77
	p-value	0.341		0.348			0.018			0.702				

Consumption categories: High: 4-7 times per week; Moderate: 1-3 times per week; Low: less than ones per week.

Total fish consumption frequency did not differ between the regions (p = 0.702) and sexes (p = 0.341) and was not associated with mother's education (p = 0.348). The fish consumption frequency was highest among participants with fathers who have a primary school education (p = 0.018). We could not relate the consumption frequency of different fish types to demographic characteristics due to the low number of participants consuming fish.

### Effect of socio-demographic characteristics on calculated meat and fish consumption quantity per week

Differences in calculated consumption quantity per week between the regions are presented in Table 3. According to ANOVA test, the quantity per week of total meat, beef, lamb, goat, and fish did not differ between the regions (p > 0.05). The highest poultry and horse meat consumptions were recorded in the East region (p = 0.012 and p = 0.034, respectively). Pork consumption was highest in the North region (p < 0.001).

**Table 3.** Comparison of recommended consumption of meat products with calculated consumption* by participants from 5 regions of Kazakhstan (n = 986). Data are presented as least squares means ± standard errors.

Meat type	Recommended consumption in Kazakhstan, g/week	Regions					P-value
		East	North	Center	South	West	
Total meat	1500 (500**)	912 ± 129	881 ± 132	1078 ± 105	983 ± 120	1051 ± 117	0.204
Beef	384	299 ± 50	258 ± 51	323 ± 41	320 ± 47	267 ± 46	0.300
Poultry	307	180^a^ ± 47	167^a^ ± 48	247^bc^ ± 38	203^ac^ ± 44	284^b^ ± 43	0.012
Horse	297	109^a^ ± 38	119^a^ ± 39	156^ab^ ± 31	126^a^ ± 35	194^b^ ± 34	0.034
Lamb	192	197 ± 36	157 ± 37	216 ± 29	203 ± 34	187 ± 33	0.314
Goat	-	73 ± 17	37 ± 18	67 ± 14	63 ± 16	54 ± 16	0.177
Pork	105	56^a^ ± 21	143^b^ ± 22	69^a^ ± 17	67^a^ ± 20	65a^a^ ± 19	0.001
							
Fish, total	269	134 ± 28	116 ± 28	125 ± 23	109 ± 26	117 ± 25	0.836

* Consumption (g/week) was estimated as (reported times per week * 150) as each serving of unprocessed meat and fish in Kazakhstan is 150 g.

** Recommended consumption by World Cancer Research Fund recommendations (WCRF, 2018).

Total meat consumption was similar between male and female participants (p = 0.190) and was not associated with mother's (p = 0.398) and father's (p = 0.200) education. Similarly, beef, lamb, goat, and fish consumption were not associated with any of the demographic factors (p > 0.05 for all). Horse consumption was highest among girls (p = 0.020). Poultry consumption was lowest among participants with fathers with a college or trade education (p = 0.021), whereas pork consumption was higher among participants with fathers with a primary school education (p = 0.050). No other significant associations were observed.

### BMI

No differences in BMI in relation to beef (p = 0.441), horse (p = 0.099), goat (p = 0.220), poultry (p = 0.131) and fish (p = 0.498) consumption frequency were detected. Frequent lamb consumers had lower BMI (p = 0.047), and frequent pork consumers had higher BMI (p = 0.05).

## Discussion

Self-reported frequency consumption is a common method to assess dietary habits (Thompson and Subar, 2017). In the present study, we used this method to conduct a survey at the national level on a sample of young Kazakhstani consumers from five regions. We focused on meat consumption because meat eating in Kazakhstan and other countries with nomad culture is identified with cultural norms. We also included the frequency of fish consumption because fish consumption is associated with a healthy diet.

Meat is a good source of a wide range of nutrients such as proteins with the optimal profile of essential amino acid, easily absorbed haem iron, zinc, selenium, and vitamins B. However, health risks have been associated with the high consumption of red meat and processed meat products (Wolk, 2017). Frequent consumption of meat, especially processed meat, has been consistently associated with an increased risk of type 2 DM in prospective studies (Song et al., 2004; Würtz et al., 2021). Moreover, processed meats might lead to high sodium intake, which increases risks for high blood pressure, heart disease, and stroke (Delgado et al., 2021; Woodruff et al., 2021). To minimize the risk of development of type 2 DM, authorities in many countries suggest healthy diet and regular physical activity (Schulze and Hu, 2005). Thus, the EAT-Lancet report in 2019 called for a decrease in animal-based foods (Willett et al., 2019). According to the Nordic Nutrition Recommendations (NNR, 2012), the consumption of red meat should be limited to an average intake of 500 g/week. These recommendations are in line with the World Cancer Research Fund recommendations (WCRF, 2018) and are based on the scientific evidence regarding associations between red meat consumption and the risk of colorectal cancer development. In Kazakhstan, meat is an important part of the traditional diets, and the newest recommendations are 1500 g/week and 78.4 kg/capita (On the approval of scientifically based physiological norms of food consumption, 2016). Total meat consumption among the participants was lower than recommended consumption in Kazakhstan but almost two-fold higher than the World Cancer Research Fund recommendations (WCRF, 2018). From 1997 to 2019, meat consumption in Kazakhstan increased by 15% (Jia and Zhen, 2021). The incidence of type 2 DM in Kazakhstan is also constantly growing (Beisembinova et al., 2021; Supiyev et al., 2016). A possible association between increased meat consumption and developing DM in Kazakhstan remains to be investigated.

Vegetarianism is a growing movement that gains popularity among adolescents and young adults. In the present study, only 0.6% of participants reported that they never consume meat, although the general prevalence of vegetarians in Asia is accounted for 19% (Hargreaves et al., 2020). Nomad diets have been unique to Kazakhstan culture, and are still followed by a large portion of the population (Aljanova et al., 2016). Approximately 24% of the participants in the present survey reported consuming meat every day, with beef and poultry being most commonly consumed. Horse and lamb meat were also consumed frequently in Kazakhstan, although the majority of the consumers from other countries are only occasional consumers of these meat types (Belaunzaran et al., 2015; de Andrade et al., 2016). Most consumers in the present study did not consider pork as their preference. The low pork consumption might be primarily related to the religious restrictions. In the present survey, we did not intend to investigate the impact of ethnicity on the preferences for meat, but we assume that pork-eaters mainly consist of the Christian population in Kazakhstan.

In the studied population, male participants consumed horse and lamb meat less frequently compared to female participants. No other sex-related differences in meat and fish consumption were observed. In contrast, other studies reported that women consume less meat compared to men and are more ready to reduce meat consumption (Beardsworth et al., 2002; Prättälä et al., 2007).

We found some significant associations between meat and fish consumption frequency and parental education. However, these associations seem to be random and difficult to explain. Generally, education level was negatively associated with meat consumption in other adult populations, including but not limited to French-speaking Switzerland (Marques-Vidal et al., 2018), Germany (Koch et al., 2019), and French-speaking men in Montreal, Canada (Trudeau et al., 2019). In contrast, a study on dietary habits of children from Moscow and Murmansk suggested that children of mothers with higher education consumed meat more frequently than the children of mothers with lower education (Aleksandrov et al., 2014). These differences between countries might be due to different nutrition recommendations and general meat image; while in Western Europe meat reduction is gaining in popularity, Kazakhstan and Russia continue considering meat as an important part of a healthy diet.

According to the Dietary Guidelines for Americans and Nordic Nutrition Recommendations (NNR, 2012), the optimal consumption of fish and shellfish is two to three times a week. Among Swedish male adolescents, 20% reported eating fish more than once a week (Åberg et al., 2009). In the present study, only 8.6% of the participants in our study reported a consumption according to these recommendations, whereas 77% of participants eat fish and seafood more seldom than once per month. In the neighbouring countries Kyrgyzstan and Uzbekistan, fish consumption is even lower, whereas inhabitants in China and Russia consume more fish than in Kazakhstan (York and Gossard, 2004). N-3 fatty acids eicosapentaenoic acid (EPA) and docosahexaenoic acid (DHA) are essential components of a healthy diet and their beneficial effects are well recognized (Campoy et al., 2012; Simopoulos et al., 1999). Oily fish is the best source of EPA and DHA. Moreover, fish is a major dietary source of vitamin D. According to Gromova et al. (2020), approximately 60% of the adult population of Kazakhstan might suffer from vitamin D deficiency. Even though vitamin D can be synthesized in human skin after exposure to sunlight, dietary vitamin D is important particularly during dark months (Kulie et al., 2009). Moreover, the high iodine levels in fish and seafood might decrease the risk of iodine deficiency, which remains highly prevalent in Kazakhstan (Kuanyshbekova, 2003).

Our data indicate that meat and fish consumption was homogeneous across regions, sex and age. Thus, it is difficult to target to a particular group to improve dietary habits and work should be initiated for general young population in Kazakhstan. It should also be emphasized that the participants in the present study did not have any education on nutrition and health during the study.

Interestingly, we found that BMI was highest among participants who reported frequent pork and low lamb consumption. Many studies demonstrated positive associations between meat consumption and risk for overweight and obesity (Wang and Beydoun, 2009). However, no difference in BMI between pork, beef and chicken consumers was previously detected (Murphy et al., 2014). Observed in the present study differences in BMI between participants with high and low pork consumption should be interpreted with caution because this type of meat is not a part of typical diet in Kazakhstan. Observed differences in BMI in relation to lamb consumption have never been previously reported.

Our study has several strengths. The majority of previous studies was focused on overall meat consumption, without considering the type of meat. Because of the existing differences in meat preferences, we considered frequency consumption of different meat types. Another strength was the geographical spread of participants over Kazakhstan. There were also potential uncertainties in our results. There are several limitations associated with dietary assessment, including self-reported food frequency questionnaires. We are also aware that consumption quantities of meat and fish calculated from shelf-reported food frequency provide only an approximate estimation of factual food consumption. Finally, the results are limited to the younger population in Kazakhstan and may be inapplicable to the general population.

## Conclusions

Calculated total meat consumption among the university students in Kazakhstan was lower than recommended consumption in Kazakhstan (1500 g per week), but almost two-fold higher than World Cancer Research Fund recommendations (500 g per week). Approximately 24% of the participants reported consuming meat every day, with beef and poultry being most commonly consumed. Another relevant point is low fish consumption among the participants. Only 8.6% of the participants in our study reported consumption in line with the recommendation of fish consumption of approximately 270 g per week in Kazakhstan. Our data indicate that meat and fish consumption is fairly homogeneous across regions and sex. Thus, it is difficult to target a particular group to improve dietary habits and work should be initiated for the general young population in Kazakhstan. The next step is to describe the major drivers of current consumption habits in Kazakhstan and identify its relationship with health of general population. Moreover, the solid scientific basis of appropriate dietary recommendations for meat and fish consumption should be established. Policies to promote healthy food consumption in Kazakhstan are needed to prevent the health risks associated with low intake of the n-3 PUFA and vitamin D, and high intake of meat. It is also essential to promote healthy dietary habits, for example through the introduction of training programmes and appropriate advertisements of healthy food.
